# Exploring new pharmacology and toxicological screening and safety evaluation of one widely used formulation of *Nidrakar Bati* from South Asia region

**DOI:** 10.1186/s12906-015-0635-2

**Published:** 2015-04-16

**Authors:** Afria Zaman, Md Shamsuddin Sultan Khan, Lucky Akter, Sharif Hossain Syeed, Jakia Akter, Abdullah Al Mamun, Md Ershad Alam, Md Ahsan Habib, Md Abdul Jalil

**Affiliations:** 1 grid.443057.1Department of Pharmaceutical Sciences, University of Development Alternative, Dhanmondi, Dhaka 1209 Bangladesh; 2 grid.443051.7Department of Pharmaceutical Sciences, University of Asia Pacific, Dhaka, Bangladesh; 3 grid.443034.40000000088778140Department of Pharmaceutical Sciences, State University Bangladesh, Dhaka, Bangladesh; 4 grid.443032.2Department of Pharmaceutical Sciences, Stamford University Bangladesh, Dhaka, Bangladesh; 5 Department of Pharmaceutical Sciences, Bangladesh University, Mohammodpur, Dhaka Bangladesh

**Keywords:** *Nidrakar Bati*, CNS, Metabolic, Analgesic, Anti-inflammatory, Neuropharmacological, Psychopharmacological, Toxicity, Gastrointestinal

## Abstract

**Background:**

*Nidrakar Bati (NKB)* is an herbal remedy consisted with seven medicinal herbs widely used to cure *Somnifacient* (sleeping aid) in South Asia as Ayurvedic medicinal system. In the present study, pharmacological and toxicological effects of this medicine was investigated in mice to validate the safety and efficacy of the herb.

**Methods:**

Organic solvent extracts NKB were prepared using maceration method. Effect of extracts on the central nervous system was evaluated using hypnotic activity assay. Effect of the extracts on metabolic activity, assessing involvement of thyroid was conducted using hypoxia test. analgesic and anti-inflammatory activities were assessed in mice using acetic acid induced writhing, formalin induced paw edema, xylene induced ear edema assays. Anxiolytic activity was performed using plus maze, climbing out and forced swimming tests. Effect of the extracts on psychopharmacological effect was carried out using locomotor activity tests (open field, Hole-board and Hole-cross tests). Neuropharmacological effect of the extracts was performed using motor coordination (rotarod test). Toxicological potential of the extract was evaluated using gastro-intestinal activity (gastric emptying and gastrointestinal motility tests).

**Results:**

The studied formulation reduced the CNS stimulant effects dose independently. In the hypoxia test, only a dose of 100 mg/kg of NKB decreased the survival time. Orally administration of the NKB (200 and 400 mg/kg) produced significant inhibition (*P* < 0.01) of the acetic acid-induced writhing in mice and suppressed xylene induced ear edema and formalin-induced licking response of animals in both phases of the test. NKB showed locomotor activity (p < 0.05) both in higher and lower doses (100 and 400 mg/kg). NKB increased the total ambulation dose dependently (p < 0.05). NKB, at all tested doses (100, 200 and 400 mg/kg) increased some locomotion activity parameters (ambulation, head dipping and emotional defecation) in hole board test. At higher doses (200 and 400 mg/kg), NKB showed a significant increase in hole cross test. NKB showed an increase in the time on the open arms of the maze at low to medium doses (100 and 200 mg/kg). When using the Rotarod method, NKB showed a considerable increase on motor coordination of the mice. NKB produced marked gastric emptying effect and decreased gastrointestinal motility in mice at low dose.

**Conclusions:**

NKB demonstrated various pharmacological effects and toxicological effects due to presence of several herbs in the formulation those are not closely fit for the effect of CNS depressants.

## Background

Herbs are useful for the treatment of different diseases that affect human and animal. They are good starting points for the discovery of bioactive molecules for drug development. Ethnomedicinally, herbs are used for pain relief, wound healing and abolishing fever that bring useful information to identify a wide range of compounds to develop new therapies for cancer, hypertension, diabetes and anti-infective medicines [1]. Herbs contains not only useful medicinal constituents but may also harmful substances [2]. Over 1500 herbal products sold are nutraceuticals which are exempt from extensive preclinical efficacy and toxicity testing by the US FDA in 2003 [3].

Nidrakar Bati (NKB) is widely prescribed for a variety of conditions, particularly insomnia. In South Asia region, people of Bangladesh, India, Pakistan, Srilanka, Nepal and Bhutan use NKB for insanity, insomnia, swooning, and headache. It is relatively safe and with overdose may result in death and addicting and abuse by patients with addiction disorders. NKB is normally used for the CNS diseases (Insomnia). This drug has some other effects on CNS and peripheral nervous system. This herbal medicine has shown some other pharmacological and toxicological effects as reported by patients. This make concerns about potential harmful as well as useful effects of this herbal medicine to justify the acute high-dose effects, chronic low-dose toxicity and specific cellular, organ and system-based toxicity assays. Despite the growing market demand for this herbal medicine, there are still concerns associated with not only its use, but its safety. Caution must be used when prescribing NKB to patients. This raises alarming condition on its safety and implications for its use as medicine. Toxicity testing makes know the risks that may be associated with use of multiple herbs to avoid potential harmful effects when used as medicine.

NKB is produced using many medicinal plants in many doses. The formulation of NKB is varied from region to region. Seven medicinal plants of *Nardostachys jatamamsi DC., Rauvolfia serpentina (L.) Benth. ex Kurz.*, *Canscora decussata (Roxb.) Schult.*, *Phyllanthus Emblica Linn.*, *Datura Stramonium Linn.*, *Cannabis Sativa Linn.*, *Acorus Calamus Linn.* (Table 1) were used to formulate this herbal medicine. *Nardostachys jatamamsi DC.* is an erect perennial herb grows up to 60 cm in height and used as folk medicine for pitta, vata, burning sensation, insanity, epilepsy, asthma, bronchitis, headache, inflammations, colic, flatulence, hepatitis, kidney diseases, low back pain, hypertension, graying or falling of hair and general debility. *Rauvolfia serpentina (L.) Benth. ex Kurz.* is a small erect shrub grows up to 60 cm in height. It is used as folk medicine for vitiated kapha, vata, hypertension, insanity, epilepsy, insomnia, wounds, fever, colic and urinary retention. *Canscora decussata (Roxb.) Schult.* is a small erect annual herb grows up to 50 cm in height and used as folk medicine for vitiated kapha, insanity, epilepsy, nervine debility, pain, skin diseases, ulcer, worms, abdominal disorders and general debility. *Phyllanthus emblica Linn.* is a medium sized deciduous tree grows up to 25 meters in height. It is used as folk medicine for tridoshas, constipation, stomatitis, jaundice, disorders of vision, fever, cough, wheezing, cardiac disorders and general weakness. *Datura Stramonium Linn.* is an erect spreading annual or biennial plant grows up to 2 meters in height and used as folk medicine for vata, kapha, arthritis, cough, asthma, muscle spasm, fever, ulcer, skin diseases, lumbago, sciatica and dandruff. *Cannabis Sativa Linn.* is an erect annual herb, grows up to 5 m in height and used as folk medicine for vitiated vata, pain, Insomnia, abdominal disorders, cough, insanity, erectile dysfunction, and inflammation. *Acorus Calamus Linn.* is a rhizomatous, perennial semi aquatic plant grows up to 40 cm in height and it is useful for the treatment of vata, kapha, insomnia, insanity, mental diseases, epilepsy, mania, stomatitis, hoarseness of voice, colic, flatulence, amenorrhea, dysmenorrhea, neuropathy, renal calculi, cough, inflammation, arthritis, kidney diseases, hemorrhoids, skin diseases and general debility.

**Table 1:** **Medicinal plants cross checked by “The Plant List” (** **www.theplantlist.org** **) used in the formulation of Nidrakar Bati and authenticity of the plant specimen by Bangladesh National Herbarium (BNH), Mirpur, Dhaka**

**Plant name (local and English)**	**Botanical name**	**Family**	**Quantity used in NKB (%)**	**Parts used**	**Chemical components**	**BNH accession number**
Jatamamsi, English : Indian night shade	*Nardostachys jatamamsi DC.*	Valerianaceae	35.71%	Rhizome	Terpenoids, alkaloids, neolignans, lignans, coumarins, nardal, jatamansic acid, BR606 and nardin	32357
Sarpagandha, English : Indian snake root. Serpentine root.	*Rauvolfia serpentina (L.) Benth. ex Kurz.*	Apocynaceae	17.85%	Root	Yohimbine, reserpine, ajmaline, deserpidine, rescinnamine, serpentinine.	36788
Sanhkapushpi, English : Canscora	*Canscora decussata (Roxb.) Schult.*	Gentianaceae	17.85%	Whole plant	Shankapushpine, Ceryl alcohol, β-Sitosterol, Evolvin, Betaine, Pentatriacontane, Triacontane, Glycoalkaloid, Gentianine, Mangiferin, Triterpenes – β – amyrin,Taraxerol, Taraxerone, Tannins & resins.	33065
Amalaki, English : Indian Gooseberry	*Phyllanthus emblica Linn.*	Euphorbiaceae	7.14%	Fruit rind	Ascorbic acid, ellagitannins, punicafolin, phyllanemblinin A, phyllanemblin, flavonoids, kaempferol, ellagic acid and gallic acid.	26450
Dhattura, English : White Thorn apple	*Datura stramonium Linn.*	Solanaceae	7.14%	Seed	Tropane lkaloids atropine, hyoscyamine and scopolamine	30120
Bhang, English : Indian hemp	*Cannabis sativa Linn.*	Cannabinaceae	7.14%	Seed	Tetrahydrocannabinol , Cannabidiol, α-Pinene, Myrcene, Linaloo, Limonene, Trans-β-ocimene, α-Terpinolene, Trans-caryophyllene, α-Humulene, Caryophyllene-oxide	33260
Vacha, English: Sweet flag	*Acorus calamus Linn.*	Araceae	7.14%	Rhizome	Asamyl alcohol, Eugenol, Asarone, Acorin (Glucoside), Starch and Tannin	35710

The molecular mechanism of action of NKB is still unknown. About 10% of herbal product in the world is truly standardized to known active components. Strict quality control measures are not always diligently adhered to safety [4]. Very little is known about the active and toxic constituents of majority of the herbal products. Herbal medicines are not subjected to the same regulatory standards as orthodox drugs in terms of efficacy and safety in developed countries of USA, UK, Europe and Canada. There is an increasing concern to use safe herbal medicine. Many herbs are responsible of adverse reaction during receiving allopathic drugs. Most of the effects were found in pregnant women and children. The increasing knowledge on herbs provides a scientific definition to design a rational herbal medicine and filter the compounds that produce toxicities. Generally, patients do not share the information of their herbal medicine with the physician. This behaviour leads lack of surveillance to monitor the herbal medicines and proper guideline for combined use of modern drugs and herbal medicine. As a result, the herbal medicine may show complementary activity or adverse herb-drug interactions. Therefore, the present study was performed to screen the pharmacological effects, toxicological effects and optimization of these effects for NKB by using in vivo study.

## Methods

### Drugs and chemicals

The NKB was collected from Sree Kundeswari Aushadhalya Ltd., Chittagong 4342, Bangladesh. The powdered tablets were dissolved to make solution to measure optimal dosage. The drug was administered orally at a dose of 100 mg/kg body weight. Barium sulphate (BaSO4), Carboxy methyl cellulose (CMC) was purchased from Merck, Germany*.* Pentobarbital were bought from Sigma Company (St Louis, MO, USA). Pentobarbital were dissolved in saline: tween 80 (9:1). All the solutions were freshly made on the day of testing and administered to a final volume of 10 mL/kg body weight of mice.

### Animals

Male (six weeks old) Swiss-Webster strain mice (20–40 gm body weight) were purchased from Animal House Department, ICDDR’B, Bangladesh. Animals were maintained under constant temperature (24 ± 2°C), 12 h light–dark cycle, relative humidity 40–70%, fed with food (mouse chow, BCSIR, Bangladesh) and water ad *libitum* and fasted overnight (18 h) before the day of the experiment. All screening procedures carried out according to the guidelines of international guideline outlined in the guide for the care and use of laboratory animals (National Research Council) [5]. The animal handling protocol of the present study was submitted to the institutional animal ethics committee, “Experimental Animal Ethics Committee, UODA” for evaluation and Animal experiment protocol was approved by the committee (approval Reference number: e/m-7PHA040294).

### Industrial manufacturing process of NKB

The batch number of collected NKB was 02-P-04\09\09-E10\11, DAR no. 58A-152 and each tablet (500 mg) contained 100 mg *Nardostachys jatamamsi DC.,* 50 mg *Rauvolfia serpentina (L.) Benth. ex Kurz.,* 50 mg *Canscora decussata (Roxb.) Schult.,* 20 mg *Phyllanthus emblica Linn.,* 20 mg *Datura stramonium Linn.,*20 mg *Cannabis sativa Linn.,* and 20 mg *Acorus calamus Linn.* The whole/partial part of these plants are dried and attach it with 44 X 32 inch art paper and submitted to identification purpose in the Herbarium and specimen voucher no. is mentioned in the Table 1.

*The manufacturing process of tablet is depicted following:*

#### Method of extraction

Most of the plants used for NKB production purposes are cultivated, that is, grown on farms. Some, however, may be collected from the wild. After harvesting and collecting, the plants were cleaned. Unnecessary parts were removed before. Plants were shed dried first. Then put in the large room with proper air permeability for several weeks at room temperature and 65% relative humidity. Fresh plants placed on a conveyor belt to pass under the warm air for 2.5 to 6 hours, and the temperature of the drying air ranges from 40 to 80°C. After drying, plants were packaged for next processing. Dried herbaceous plants were generally compressed into bales weighing from 60 to 100 kg (13 to 220 pounds), which were kept in plastic bags. Materials that were not dried and processed properly, such as roots and bark, were placed in sacks. Dried plant materials were stored under controlled humidity and temperature in sealed container. The sacks were passed into processing facility to remove impurities.

Sand and iron-containing metals were removed pneumatically and magnetically, respectively. Next, machine sorts out plant parts according to size. Shredded materials were used for extraction. Large size particles were treated with additional crushing and sieving. Organic solvent extraction was used to separate the plant constituents. The plants were first ground and then thoroughly mixed with a solvent methanol in the large aluminium container. Dissolved active ingredient of the plant was used. Standard filtration was applied exchange to separate impurities from the plant extract. Rota evaporator was used to thicken the extract. Extracts were completely dried using cabinet vacuum dryers with the goodness of moisture and stability. Then obtained powdered extract was preserved since it has no ability to contaminate with microbes.

#### Preparation of the final formulation

NKB Tablets were made using magnesium stearate and gums. The quantity of excipients were less than 50% of the weight of the finished product.

#### Quality control of extracts

Several methods were performed to determine the quality of the extract. Physical characteristics of the extract, pH, solubility, total solids content, ash content, and particle size were determined using standard testing procedure mentioned in the Table 2. Thin Layer Chromatography (TLC) was used to identify the phytoconstituents from the extract. Microbial contamination was tested by the disc diffusion method. The extract concentration in the tablet was determined by standard mathematical analysis, i.e. tablet containing 500 mg of extract standardized to 50 percent that will be 250 mg of extract.

**Table 2:** **Physicochemical parameter of NKB extract and tablet**

**Physicochemical**		**Test**	**Results**	**Spec.**
Extract appearance		Visual performance	Brownish, Aromatic odour, Tasteless	NA
pH		pH Meter	5.23	NA
Moisture content(100-105°C)		Moisture content analyzer	2.2%	NMT 1%
Solubility	Water soluble extractive	Add water Q.S.	18%	NMT 16%
	Alcohol soluble extractive	Add isopropyl alcohol Q.S.	20.3%	NMT 9%
Total solids content		Microwave Solids Analyzers (CEM LabWave 9000)	0.69	NMT 1%
Ash content	Total ash	Wt. of sample and wt. of original sample ratio is determined	6.23%	NMT 7%
	water soluble ash	As above	5.50%	
	Acid insoluble ash	As above	0.88%	NMT 1%
	Sulphated ash	As above	6.13%	
Particle size/shape		Microscopic analysis	12-14.5 cm length, 1–2 cm thick; Cylindrical and branched	NA
Foreign matter (Residual solvents, Herbicides, pesticides, Microbial contamination)		Disc Diffusion Assay	0.75%	NMT 1%

The specification mentioned in the table obtained from quality control lab of Sree Kundeswari Aushadhalya Ltd.-Ctg. The results found after tested were compared to this specification.

#### Thin layer chromatography (TLC) of methanolic Extract and NKB tablet

Standard size TLC plates were cut into small pieces as Length – 7 cm, Width – 2.5 cm, Labeling space – 1 cm from both upper & lower side. Both the plant extract and NKB tablet were used for chromatographic separation by TLC method. TLC plates in Iodine chamber showed a significant results in 9:1, 8:2, 7:3 and 6:4 mobile phase solvent ratio (n-hexane : ethyl acetate) and found spots in TLC with R_f_ values 0.65, 0.69, 0.68, 0.67 and indicated different verities of compounds.

### Treatment

Mice were divided into 10 groups. Each group had ten mice. All experimental extract and standard medicine were administered orally. Mice were received 10 mL/kg normal saline as vehicle and served as a negative control for the NKB. NKB administered mice groups received low, medium, high doses of 100, 200 and 400 mg/kg, respectively. All experiments were conducted between 8:00 and 13:00 every day to avoid any temporal factor. Each animal was used for only one experimental condition.

#### Hypnotic activity (CNS effects)

##### Pentobarbital sleeping time test

NKB was administered orally (100, 200, 400 mg/kg) 60 min afterwards mice were administered with sodium pentobarbital (45 mg/kg, oral). The time from the loss of rightness reflex to awakening (duration of sleeping) were calculated (in minutes) for each animal [6]. Mice were given a single oral dose of the vehicle, diazepam (2 mg/kg) as the reference drug. These treatments were carried out 60 min before challenging the animal with oral dose of pentobarbital (45 mg/kg). To investigate the possible mechanism involved in the hypnotic activity of *NKB*, the animals were pretreated with flumazenil (10 mg/kg), antagonist of GABA_A_–benzodiazepine receptor*.*

#### Metabolic activity

##### Hypoxia test

Mice were placed in an air tight empty glass jar of 300 mL capacity attached with an electronic watch, and the onset of convulsion time was recorded [7]. The hypoxia time was recorded after 2 hr.

#### Analgesic and Anti-inflammatory activity

##### Acetic acid induced writhing

The *NKB was* evaluated for analgesic activity in mice using acetic acid induced writhing [8] tests described below. Mice were injected intraperitoneally with 0.6% aqueous acetic acid (10 ml/kg) 30 min after oral administration of NKB (100, 200, 400 mg/kg) or vehicle (Saline, 10 ml/kg). The reference group was given Diclofenac sodium (25, 50, 100 mg/kg). The number of writhing movement (painful Muscular contraction) of each mouse was counted for 10 min, starting from 15 min after the injection of acetic acid. The average number of writhes and the percent protection were calculated as following:

$$ \mathrm{Percentage}\kern0.5em \mathrm{analgesic}\kern0.5em \mathrm{activity}=\frac{\mathrm{N}-{\mathrm{N}}^{\mathrm{t}}}{\mathrm{N}}\times 100 $$

Where N is the average number of stretch of control animals per group And N^t^ is the average number of stretching of treated animals per group.

##### Formalin induced paw licking test

The applied method was similar to previously described process of Correa and Calixto [6]. Eighty microgram of 1% formalin solution (Sigma) was administered subcutaneously in the right hind paw for the induction of pain. The duration of time in licking the injected paw was monitored. After 5 min of formalin injection, the first nociceptive response (first phase: neurogenic) was counted. After twenty min of formalin injection, the second phase (inflammatory) was recorded. The mice were pretreated with NKB at 1 h, before formalin injection, and the responses were observed for 30 min.

##### Xylene-induced ear oedema test

Xylene (0.03 ml) was injected in the right ear (anterior and posterior surface) after 30 min of the injection (i.p.) of NKB. Dexamethasone (50, 150 and 300 mg/kg, b.w.) was used as positive control. The untreated mice group was considered as control. Mice were killed after 2 h xylene injection. The ears were removed from NKB treated, positive control and control groups of mice. The subtracted weights from the experimented groups were calculated [9,10] using following formula:

$$ Oedema\  reduction\  inhibition\;\%=\frac{Rt-Lt}{Rc-Lc}\times 100 $$

Where Rt = mean weight of the right ear plug of the treated animals; Lt = mean weight of the left ear plug of the treated animals; Rc = mean weight of the right ear plug of the control animals; Lc = mean weight of the left earplug of the control animals.

#### Psychopharmacological activity

##### Open field test

A white colored open floor of 100 × 100 cm divided by red lines into 25 squares of 20 × 20 cm [11]. The place was fenced (50 cm) with white color. The test was conducted at the normal day light maintaining same intensity of light. Each mouse was kept in the center of the open field, and its behavior was observed for 5 min. Some locomotion parameters total, peripheral and central locomotion were determined according to the total number of squares crossed, the number of outer squares adjacent to the walls crossed, How many leanings (one to two paws touching the wall), parenting, grooming (face cleaning, paw licking, fur licking, scraping and also rubbing), respectively [12]. Locomotion or Ambulation was determined as the number of times crossing the square with all four limbs, if peripheral squares it is peripheral ambulation, if it is central squares then it is central ambulation. Also other noted parameters were standing up behavior (Immobilization) time and Emotional defecation (Urination).

##### Hole board test

The three doses of NKB (100, 200 and 400 mg/kg) was administered orally 60 min before test and standard group was treated with diazepam (2 mg/kg) intraperitoneally 30 min before test. The control group was treated with normal saline, before the test of 60 min. Initially mouse was kept in the edge of the board (constructed with 16 × 3 cm in diameter). the number of ambulation (the number of holes passed), head dipping and number of fecal boluses excretion were taken as the measurement for 30 minutes prior and post 30, 60, 120 and 240 minutes [13].

##### Hole cross test

A box (30 × 20 × 14 cm dimension) with a hole of 3 cm in diameter and 4.5 cm in height from the floor was constructed. Spontaneous movement of the animals through the hole from one to the other was counted [14,15]. The observation was conducted 30, 60, 120, 180 and 240 minutes after oral administration of test drugs.

#### Anxiolytic activity

##### Climbing out test

Mice were kept in the cage (60 × 50 × 30 cm) surrounded by black colored. Mice were trained prior the test to climb a ladder of (6 cm long) suspended from a clamp of a retort stand (100 cm above ground). A ladder was provided and time was taken to climb of the cage. Only mice those climbed the ladder within 10s were selected for the test. This test was carried out 30 min after treatment with *NKB* and diazepam and normal saline were used as control (sec).

##### Elevated plus maze test

The three doses of NKB (100, 200 and 400 mg/kg) were administered orally 60 min before test and the standard group was treated with diazepam (2 mg/kg) intraperitoneally 30 min before test. The control group was treated with normal saline orally, 60 min before test. At the beginning of test, mice placed on the open arm facing the center of the plus maze. The time (s) spent by mice in the open and closed arms was recorded. Anxiolytic compounds reduced the mice’s natural aversion to the open arms and support exploration. Therefore, increased time spent in the open arms was defined to reflect an anxiolytic effect, in comparison with the control group [16].

##### Forced induced swimming test

Forced swimming test (FST) is usually used as Pharmacological model for antidepressant action. The mice were treated with the NKB (100, 200 and 400 mg/kg, oral), Diazepam (2 mg/kg, IP) and saline solution before 45 min of the test. The forced swimming mice exposed to passive and immobile after a period of vigorous activity (struggling) to keep their heads above the water. Swimming sessions were conducted in individual Plexiglass cylinders (40 × 20 cm) containing 20 cm of water at 24 ± 1°C. Mice were forced to swim for 6 min and the time spent in immobility during the last 5 min of 6 min observation period was recorded manually by the competent observer [17].

#### Neuropharmacological activity

##### Rotarod test

Pharmacological effects of anxiolytic drugs are tested to analysis the loss of coordinated motor movement [18]. Coordinated motor activity of NKB was assessed using rotarod test [18]. Previously trained mice adapted to rotarod apparatus (3 cm in diameter, 8 rpm) for 120 seconds and at least two times for each animal. After twenty four hours mice were injected with vehicle (saline), NKB (100, 200 and 400 mg/kg) orally and placed in instrument 1 hour later. Immediately (in seconds) dropped off mice from the rotarod was recorded up to a limit of 100 seconds [19].

#### Toxicological activity

##### Gastric emptying time

Sixteen male Swiss-Webster mice were fasted for 18 hours prior to experiments. The described method of Droppleman et al. [20] and Martinez et al. [21] was used here. Rats fasted for 24 h prior to the experiment received distilled water (10 ml/kg) and NKB (100, 200 and 400 mg/kg). One hour after, 3 ml of a semi solid test meal (based on methyl cellulose) was administered to the mice. The mice were sacrificed and laparatomized 1 h after the treatment and the stomach removed. The full stomach was weighed, opened and rinsed, excess moisture was mopped and the empty stomach weighed. The difference was subtracted from the weight of 3 ml of the test meal, and was taken as the quantity emptied from the stomach during the test period.

$$ \%\kern0.5em GE=\frac{Gastric\kern0.5em  Contents}{Total\kern0.5em  Food\kern0.5em  Intake}\times 100 $$

##### Gastrointestinal motility test

GI motility test was carried out to find the effect of the drugs on the peristaltic movement of the GI tract [22]. The mice (25 to 35 g) were fasted 18 to 24 hr before beginning of the experiment. Control group was given normal saline (10 ml/kg) orally, and Positive Group treated with antimotility drug Loperamide (3 mg/kg, p.o.) was used as the standard drug. Remaining groups were treated with NKB (100, 200 and 400 mg/kg, oral). BaSO4 milk was prepared by adding BaSO4 at 15% w/v in 0.5% CMC suspension. After 30 min of administered saline, castor oil and NKB, the BaSO4 milk was administered (5 mg/kg oral). After 15 min of administering BaSO4 milk, the animal was killed by cervical dislocation and dissected out. The dissected animals were placed on clean surface and the distance travelled (from pylorus to the ileoceccal junction) by BaSO4 milk was measured. Then GIT motility was calculated for all groups. The percent motility was calculated using the following formula:

$$ \mathrm{Percent}\kern0.5em \mathrm{Motility}=100\hbox{--} \left(\mathrm{Distance}\kern0.5em \mathrm{covered}/\mathrm{total}\kern0.5em \mathrm{length}\kern0.5em \mathrm{of}\kern0.5em \mathrm{intestine}\right)\times 100. $$

### Statistical analysis

Data are expressed as mean ± SD (Standard Deviation). The statistical significance was determined using the unpaired t test. *P < 0.05* was defined as indicative of significance as compared to the control group.

## Results

Physicochemical parameters of tablet and extract were tested using standard lab analysis protocols and obtained results were satisfactory but limited according to specifications (Table 2).

### Analgesic and anti-inflammatory activity

#### Acetic acid induced writhing

**Figure 1:**
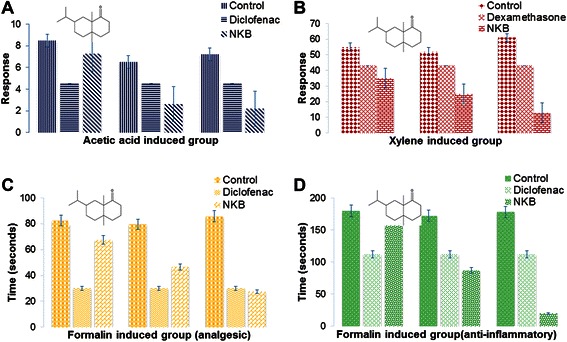
Analgesic and anti-inflammatory activity of NKB with various doses (100, 200, 400 mg/kg/day) and standard drugs diclofenac (25, 50, 100 mg/kg/day) and dexamethasone were performed in mice model. Dose dependent activity of NKB was observed that was reproduced with diclofenac and dexamethasone. Diclofenac with 25 mg/kg/day and dexamethasone with 50 mg/kg/day showed highest efficacy and the result of NKB was compared based on this activity. Three mice models were used for in vivo experiments to determine the analgesic and anti-inflammatory activity such as **(A)** acetic acid induced writhing response (p < 0.01), **(B)** xylene induced ear oedema (p < 0.01), **(C)** formalin induced –analgesic (p < 0.05), **(D)** formalin induced – inflammation (p < 0.05). Analgesic effects come from the active principle of *jatamansone (structure shown) and* 50% rhizomes ethanol extract of *Nardostachys jatamansi* DC. Also, flavonoids from *Rauvolfia serpentina (L.),* ascorbic acid, tannins and polyphenolic compounds, hydro-methanolic extract, water fraction of fruits butanol extract of *Phyllanthus emblica L.*, ethanolic extracts of *Datura stramonium Linn.,* active principles of cannabigerol, cannabichromene, cannabidiol, delta-9 tetrahydrocannabinol, delta 9 tetrahydrocannabivarin of *Cannabis sativa Linn.,* phenolic compounds, essential oil and alcoholic extract of the rhizomes of *Acorus calamus Linn.* are responsible for the anti-inflammatory activity. Since *Canscora decussata (Roxb.) Schult.* has no analgesic activity till now and hence it is not suggested to use in NKB formulation. Although *Datura stramonium Linn.* has anti-inflammatory activity, it is not recommended due to alkaloids atropine and scopolamine for diarrhea, vomiting, hypoactivity, and liver weight loss*. Phyllanthus emblica L.* has strong cytotoxic activity (Table 3) due to phytosterol and phenolic compounds and hence minimum quantity is recommended in NKB formulation.

The different doses of the NKB (200 and 400 mg/kg) decreased writhing response induced by acetic acid administered intraperitoneally to mice (Figure 1) in a dose-dependent manner. The standard analgesic diclofenac sodium (25 mg/kg, b.w.) reduced the abdominal constriction to 90%.

#### Xylene-induced ear oedema test

There were significant differences between the NKB and control groups after treatment (Figure 1).

#### Formalin induced paw licking test

The nociceptive responses were quantified by the time of paw licking after the formalin injection. The results depicted in Figure 1 showed that the NKB caused significant inhibition of both neurogenic and inflammatory phases of formalin-induced licking. Its antinociceptive effects were significantly more pronounced than diclofenac (25 mg/kg). The second phase was more reduced than first phase at the dose of 400 mg/kg of NKB.

### Psychopharmacological activity

#### Open field test

**Figure 2:**
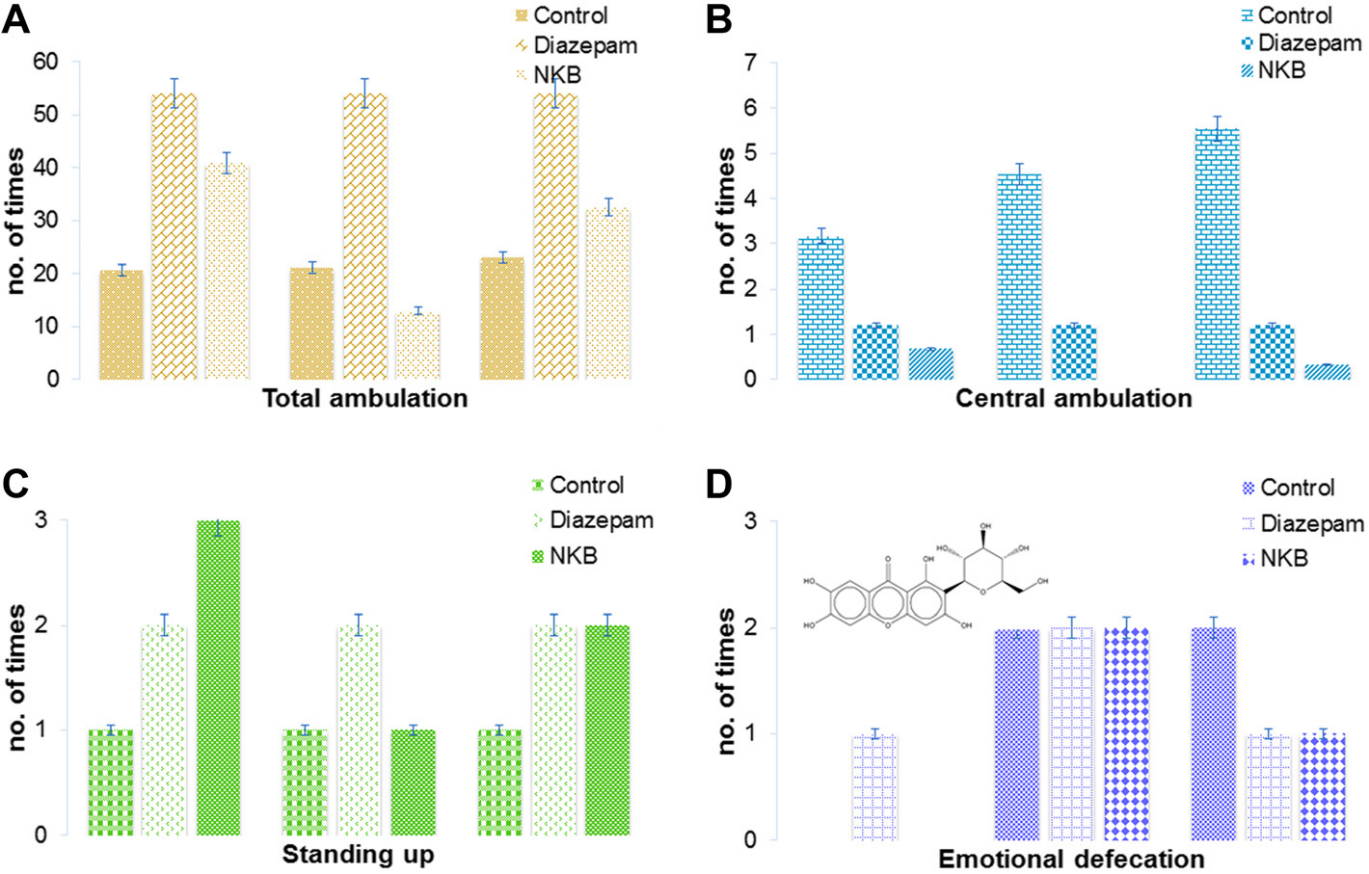
Psychopharmacological activity was determined by open field test using **(A)** total ambulation **(B)** central ambulation **(C)** standing up behaviour **(D)** emotional defecation parameters in mice. (p < 0.05). The active principles of serpentine (structure shown, **B**) of *Rauvolfia serpentina* (L.), total xanthones (structure shown, **C**) of *Canscora decussata (Roxb.)* Schult., cannabidiol (structure shown, **D**) of Cannabis sativa Linn. may be the reason for such effect.

About all doses of NKB and diazepam (3 mg/ kg) lowered the open field characteristics except total ambulation (Figure 2).

#### Hole board test

**Figure 3:**
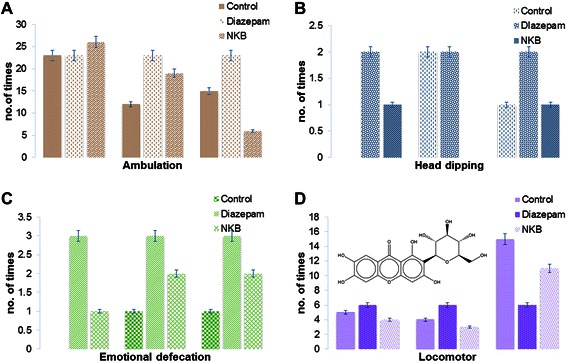
Anxiolytic activity was found in hole board test by observing ambulation **(A)** (p <0.05), head dipping **(B)**, emotional defecation **(C)** parameters and in hole cross test by locomotor **(D)** (p < 0.05) parameters in mice. This activity of NKB may be due to presence of total xanthones of *Canscora decussata (Roxb.) Schult.*, mangiferin (structure shown) of *Canscora decussata (Roxb.) Schult.,* and cannabidiol of *Cannabis sativa Linn.*

In the hole-board test, a significant reduction in the number of head dips at the doses of 100 mg/kg by oral route administration with the exception at the dose of 200, and 400 mg/kg, the NKB did not reduce the number of head dips (Figure 3).

#### Hole cross test

In the hole cross test only one group of mice showed better locomotor activity after the treatment of NKB and other groups did not response properly (Figure 3). As results 400 mg/kg of NKB decreased the locomotion that was more reduced after the treatment of Diazepam (2 mg/kg).

### Anxiolytic activity

#### Climbing out test

**Figure 4:**
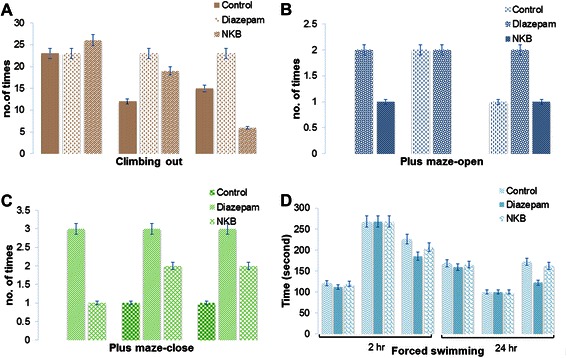
Obtained anxiolytic activity in mice performed by climbing out test **(A)**, elevated plus maze-open arm **(B)**, elevated plus maze- close arm **(C)**, forced induced swimming test **(D)** at 2 hr and 24 hr. NKB produced dose depending activity according to 100, 200 and 400 mg/kg/day. The result of the diazepam (2 mg/kg/day) was used as standard to determine the anxiolytic activity of NKB (p < 0.05). The plant presence in the NKB formulation named *Canscora decussata (Roxb.) Schult., Canscora decussata (Roxb.) Schult.* And *Cannabis sativa Linn.* may be the cause of this activity in mice.

In climbing test, loss of coordination was found significantly in mice with orally treated of NKB dose dependently (Figure 4). The results were better than Diazepam (2 mg/kg) treatment.

#### Elevated plus maze test

A significant increase in time spent in the open arms of maze and decrease in time spent in close arm of maze (Figure 4) were observed in the low dose of NKB (100 and 200 mg/kg). The diazepam (3 mg/kg, i.p.) positive control group, spent longer time in the open arms of the maze (Figure 4).

#### Forced induced swimming test

There are significant differences in the swimming time to exhaustion between the control group and each treatment group. The swimming time to exhaustion of the control, Diazepam and NKB groups were increased at all doses (100, 200, 400 mg/kg) after 2 h and decreased after 24 h (Figure 4). Thus, the swimming times to exhaustion of the NKB groups were significantly longer than that of the control group (*P <* 0*.*05).

### Hypnotic activity (CNS effects)

#### Pentobarbital sleeping time test

**Figure 5:**
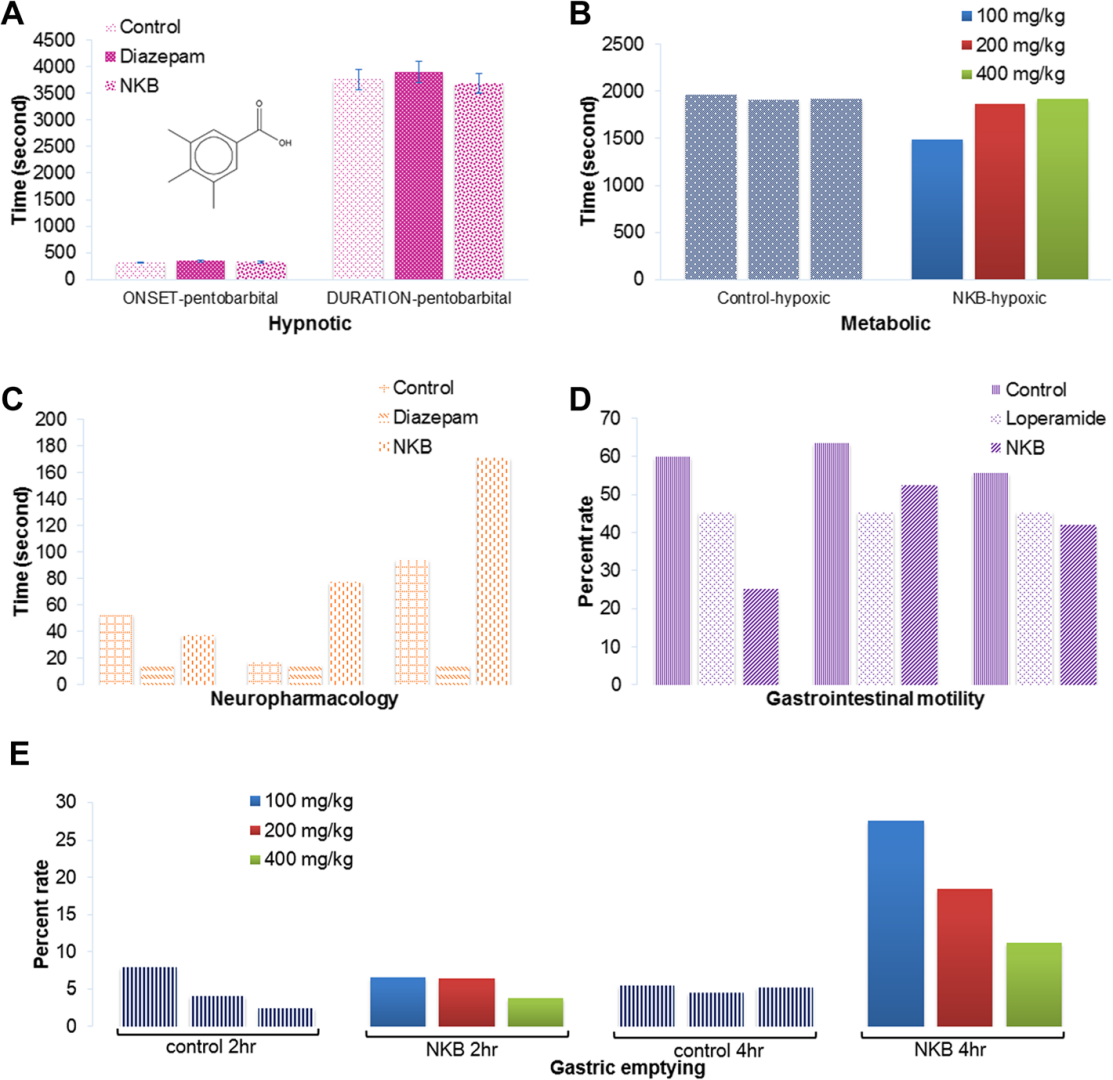
Hypnotic activity of NKB was performed using pentobarbital induced sleeping time test **(A)**. The dose of NKB was used as 100 mg/kg/day. NKB showed significant effect similar to diazepam. Metabolic study was conducted by observing survival time in seconds in the hypoxic condition **(B)**. Mice were treated low (100 mg/kg/day), medium (200 mg/kg/day) and high doses (400 mg/kg) (P < 0.05). Neuropharmacological activity was studied for NKB using rotarod **(C)**. Toxicity of the NKB was screened through gastrointestinal motility **(D)** and gastric emptying rate **(E)**. Active metabolites of *Jatamansone of Nardostachys jatamansi* DC; 3,4,5-trimethyl benzoic acid (structure shown, **A**) ester of reserpic acid, an indole derivative of 18-hydroxy yohimbine type of *Rauvolfia serpentina (L.),* mangiferin, xanthones of *Canscora decussata (Roxb.) Schult,* crude fine powder and alcoholic extraction of *Canscora decussata (Roxb.) Schult.,* cannabidiol, and cannabinol *of Cannabis sativa Linn.,* isolated constituted of the rhizomes, asarone and β-asarone of *Acorus calamus Linn*. may be the principle cause to show the hypnotic activity. However, the use of the plant *Acorus calamus Linn.* is not recommended in the NKB formulation due to its immunosuppressive activity, diarrhea and dysentery.

In the pentobarbital induced sleeping time test, the extract at a low dose 100 mg/kg and also diazepam (3 mg/kg) significantly increased sleeping time (duration) in treated group than the control group (Figure 5A).

### Metabolic activity

#### Hypoxia test

NKB treated male mice decreased the survival time in the hypoxia test at the dose 100 mg/kg. However, NKB did not show any distinction from those of the control group at the dose of 200 mg/kg and 400 mg/kg (Figure 5B).

### Neuropharmacological activity

#### Rotarod test

NKB and diazepam lowered motion harmony and function within the rotarod program at 58 and 360 minute after injections (Figure 5C).

### Toxicological activity

#### Gastrointestinal motility test

In the gastrointestinal motility test, the NKB and standard antimotility drug loperamide significantly slowed down the gastrointestinal transit of milk in comparison to control (*P* < 0.01) (Figure 5D).

#### Gastric emptying time

Increased gastric emptying time was observed in mice after 4 hour at the dose of 100 mg/kg (NKB) (Figure 5E). This effect was almost same after 2 hour at most doses of NKB (100, 200, 400 mg/kg).

## Discussion

### Screening and optimization of NKB effects

NKB is indicated as anticonvulsive, sedative, hypnosis, antidepressive, Analgesic, Anti-inflammatory, anti-nociceptive, anxiolytic, antidiarrhoeal effects and to be useful for treating nervous breakdown, nervous tension, depression, and insomnia. In this paper, we observed these mentioned activities in mice using herbal formulation of NKB. Therefore, to study the inclusive effect of this herbal remedies, the below targets were observed such as duration of sleeping, onset of convulsion time, painful muscular contraction, duration of time in licking, locomotion parameters, number of ambulation, number of fecal boluses excretion, number of head dips, loss of initiative, curiosity, spontaneous coordination, swimming capacity, percent gastric emptying and motility in mice.

### NKB relieves from the pain significantly

The acetic acid induced writhing is inversely proportionate to doses of NKB as non-narcotic analgesic property. At dose of 100 mg/kg, NKB treated male mice showed a lower effect in writhing response from 1^st^ minute to 5^th^ minute in comparison to control group. The decrease of writhing response was statistically highly significant (p < 0.01) at the 3rd minute. NKB treated mice showed higher effect in writhing response at the dose of 200 mg/kg and 400 mg/kg respectively in comparison to control group. When administered (oral) to mice, the different doses of NKB (100, 200 and 400 mg/kg) motivated the writhing response induced by acetic acid administered intraperitoneally to mice (Figure 1), in a dose-dependent manner. The inhibition percentages of writhing obtained with NKB were 14.70%, 69.41% and 74.11% respectively at the dose (100, 200 and 400 mg/kg). The NKB showed the most significant inhibitory effect (74.11% at the dose of 400 mg/kg). The standard analgesic diclofenac sodium (100 mg/kg) reduced the abdominal constriction to 47.05%. Usually, NKB is used for headache. The dose of 200 mg/kg/day is usually prescribed by the herbal medicine practitioner in South Asia.

### NKB formulation contains ample quantity of phytoconstituents that responsible for analgesic activity

The writhing inhibition increased as the dose of NKB was increased (Figure 1) in the analgesic activity performed using acetic acid-induced writhing inhibition test in mice. Herbal plants contain alkaloids, flavonoids, steroids, and tannins according to qualitative phytochemical screening [23]. Flavonoids, tannins, and alkaloids may play a role in analgesic activity primarily by targeting prostaglandins [24,25]. Besides this, herbal plants show analgesic effect due to steroids activity [25]. The results justify the traditional use of NKB as an analgesic in South Asia [26]. Percent protection of pain perception of NKB was found as 47.83% (decreased), 121.90% (increased), 40.800% (increased) at the dose of 100, 200, 400 mg/kg, respectively.

Centrally acting narcotic analgesics can inhibit both phases of pain in formalin induced paw licking effect model while peripherally acting drugs such as diclophenac sodium can inhibit the late phase (inflammation) [27]. NKB (100 mg/kg) showed higher analgesic activity in the mice at the dose of 400 mg/kg. In the contrary, NKB showed mild effect on inflammation at the dose of 100 mg/kg (*P < 0.05*).

### Anti-inflammatory activity observed in NKB

Acetic acid acts indirectly by inducing the release of endogenous mediators which stimulate the nociceptive neurons sensitive to non-steroidal anti-inflammatory drugs (NSAIDs) and opioids [28]. Acetic acid is responsible for inflammatory pain by inducing capillary permeability [29], formalin for neurogenic and inflammatory pain [30], while hot plate-induced pain points out narcotic involvement [31]. NKB (100, 200 and 400 mg/kg) administered orally on inflamed and non-inflamed paws, significantly reduced the increase in formalin induced paw edema (Figure 1C, D). The NKB showed significant anti-inflammatory activity at 100 mg/kg studied, as compared with control group. The subcutaneous application of the NKB on the non-oedematous paw reduced the inflammation of the oedematous paw. The reason may be for the transdermal absorption to produce a systemic effect. Formalin induced edema is usually correlated with the early exudates-stage of inflammation as inflammatory pathology [32]. The increased gradually inflammation may be due to the liberation of prostaglandins and kinins, which accompany leukocyte migration [33]. No written rules is found to prescribe specific amount of dose for anti-inflammatory use of NKB. Plants used in the NKB responsible for anti-inflammatory effect may be prescribed as 50 to 200 mg/kg/day (Table 3).

**Table 3:** **Effects of each plant constituent in NKB**

**Plant**	**Chemical components**	**Effects of plant extract**	**Effect of plant parts**	**Effect of active principles**
***Nardostachys jatamansi*** **DC**	Terpenoids [70], alkaloids [71], neolignans, 3 lignans [72], coumarins [73], other compounds [74,75].	50% rhizomes ethanol extract – hepatoprotective [76], liver damage protection in vivo [77], hypolipidaemic effects in vivo [78], antiarrhythmic activity [79]. Hexane extract hair growth activity	Rhizome preparation of medicinal oils, [80] to promote growth of hair [79], imparts blackness [79].	Essential oil (from the roots) fungi toxic activity [80]. Antimicrobial [81]. Antifungal [82]. Hypotensive [83] Antiarrhythmic [84] anticonvulsant activity [85]. BR606 bone sorption inhibitor for the treatment of osteoporosis and hypercalcemia [86] jatamansone (valeranone) hypotensive and tranquilizing agent, [87] antiarrhythmic and anticonvulsant agent [84] sedative activity Jatamansic acid hair growth activity
***Rauvolfia serpentina (L.)***	Alkaloids, carbohydrates, flavonoids, glycosides, phlobatannins, phenols, resins, saponins sterols, tannins terpenes different alkaloids (monoterpenoid indole alkaloid family): ajmaline, ajmalicine, ajmalimine, deserpidine, indobine, indobinine, reserpine, reserpiline, rescinnamine, rescinnamidine, serpentine, serpentinine, yohimbine. ascorbic acids, riboflavin, thiamine, niacin [88]	Same as effect of active principles	Root and rhizome high blood pressure, mental agitation, epilepsy, traumas, anxiety, excitement, schizophrenia, sedative insomnia, insanity	3,4,5-trimethyl benzoic acid ester of reserpic acid, an indole derivative of 18-hydroxy yohimbine type: natural tranquillizing effect. [89,90] Serpentine, type II topoisomerase inhibitor, antipsychotic properties [91,92] Alkaloid, ajmalicine: function of smooth muscle, prevent strokes and helps, in lowering blood pressure [93] Rescinnamine ACE inhibitor [94] Alkaloid Yohimbine selective alpha-adrenergic antagonist alpha-blocker, diabetes [95] flavonoids anti-inflammatory activity
***Canscora decussata (Roxb.) Schult.***	Alkaloids, terpenoids, phenolics phenolic compounds, xanthones, triterpenoids pentaoxygenated, hexaoxygenated, dimeric xanthones, bitter substances, oleoresin, mangiferin 1,5-dihydroxy-3-methoxy (X), 1-hydroxy-3,5-dimethoxy (VII), 1,3,5-trihydroxy-6-methoxy (II), 1,3,8-trihydroxy-7-methoxy (III), 1,8-dihydroxy-3,7-dimethoxy (XI), 1-hydroxy-3,7,8-trimethoxy (VIII), 1,3,8-trihydroxy-6,7-dimethoxy (XIII), 1,8-dihydroxy-3,6,7-trimethoxy (XII), 1-hydroxy-3,6,7,8-tetramethoxy (IX), 1,3,5,6-tetrahydroxy (XIV), 1,3,7-tetrahydroxy (XV), 1,3,6,7,8-pentahydroxy (XVI) xanthones	Petroleum extract CNS depression decreased motor activity, sedation, diminished response to external stimuli mangiferin (50 mg/kg) potentiate subnarcotic effect. mangiferin inhibition of reserpine-induced ptosis, sedation, depression of locomotor activity [96] ethanolic extract (400 mg/kg) reduce the neuromuscular coordination indicative of the muscle relaxant activity [97] crude fine powder and alcoholic extraction hypnotic activity [98] chloroform-soluble fraction of ethanolic extract antimicrobial [99] aqueous preparation activation of cell adhesion molecules [100] Extract antiinflammatory activity [101] spermicidal activity [102]	Same as effect of extract and active principles	Total xanthones CNS depression polyoxygenated xanthones antimicrobial mangiferin; Acute toxicity, CNS stimulation, mangiferin induced tremors, pilo erection, compulsive gnawing, increased motor activity mangiferin and the total xanthones (50 mg/kg) induce pentobarbitol sleeping time, transient positive inotropic effect no analgesic activity no diuretic effect (100 mg/kg) no significant effects dog’s carotid blood pressure, respiration, intestinal movements [103] magostin-3,6-di-O-glucoside and mangiferin, a C-glucoside (roots extract) protection against liver injury in vivo [104] mangiferin anti-necrosis in vivo, sedation and ptosis in vivo, potentiation of amphetamine toxicity in vivo, potentiation of dihydroxyphenylalanine (DOPA) effect in vivo, potentiation of 5-hydroxytryptophan effects in vivo, potentiation of subanalgesic dose of morphine in vivo (Bhattacharya *et al.)*
**Phyllanthus emblica L.**	Fixed oils, phosphatides, essential oils, tannins, minerals, vitamins, amino acids, fatty acids, glycosides Fatty acids: linolenic, linoleic, oleic, stearic, palmitic, myristic acids. major tannins: D-glucose, D-fructose, D-myo-inositol, D-galacturonic acid, D-arabinosyI, D-rhamnosyl, D-xylosyI, D-glucosyI, D-mannosyl, D-galactosyI residues- sugars, Emblicanin A Emblicanin B, Pedunculagin, punigluconin Other compounds: gallic acids, amlaic acid, arginine, aspartic acid, astragallin, β-carotene, β-sitosterol, chebulagic acid, chebulic acid, chebulaginic acid, chebulinic acid, corilagic acid, corilagin, cysteine, ellagic acid, emblicol, ibberellins, glutamic acid, glycine, histidine, isoleucine, kaempferol, leucodelphinidin, methionine, phenylalanine, phyllantidine, phyllemblic acid, quercetin, riboflavin, rutin, thiamin, threonine, tryptophan, tyrosine, valine, zeatin	Aqueous infusion and decoction: strong antibacterial activity [105] leaf extract antimalarial potency [106]. The chloroform soluble fraction of methanolic extract antimicrobial activity in gram positive and gram negative pathogenic bacteria strong cytotoxicity [107] hydro-methanolic extract normalize the impaired antioxidant status [108] fruit extract antidiarrheal spasmolytic activities [109] water fraction of fruits butanol extract potential anti-inflammatory [110]. Extract affected the mode of absorption as well reduces serum, aortic and hepatic Cholesterol [111] aqueous extract natural killer cell activity antibody-dependent cellular cytotoxicity [112] aqueous extract anticancer effect [113]. Extract chondroprotective Effects [114]	Fruits indigestion and constipation [115]. Decoction of leaves fever fresh fruit refrigerant. seeds cooling remedy in bilious affections nausea, fevers [116]	1,2,4,6-tetra-O-galloyl-β-D-glucose: anti-viral activity against anti-herpes simplex virus [117] ascorbic acid, tannins and polyphenolic constituents: antioxidant and free radical scavenging activity [118] tannins, corigalin and its analogue anti-atherosclerosis [119] flavonoid quercetin hepatoprotective [120] phenolic compounds The antiproliferative activity of tumor cell lines, MK-1, HeLa, and B16F10 cells phytosterol compounds cytotoxic effect in tumor and non-tumor cell lines [121]
***Datura stramonium Linn.***	Saponins, tannins, alkaloids, glycosides, alkaloids, tannins, carbohydrates, proteins, tropane alkaloids, hyoscyamine, scopolamine tigloidin, aposcopolamine, apoatropin, hyoscyamine N-oxide, scopolamine N-oxide17-20. 6â-ditigloyloxytropane, 7-hydroxyhyoscyamine [122] atropine, hyoscamine scopolamine [123] tropane alkaloids, 3-phenylacetoxy-6, 7-epoxynortropane, 7-hydroxyapoatropine, The alkaloids scopoline, 3-(hydroxyacetoxy) tropane, 3-hydroxy-6-(2-ethylbutyryloxy) tropane, 3â-tigloyloxy-6-hydroxytropane, 3, 7-dihydroxy-6-tigloyloxytropane, 3-tigloyloxy-6-propionyloxytropane, 3 phenylacetoxy-6,7-epoxytropane, 3-phenylacetoxy-6-hydroxytropane, aponor scopolamine, 3â, 6â-ditigloyloxytropane, 7-hydroxyhyoscyamine [124]. Hygrine, 3á, 6â-Ditigloyloxy-7-hydroxytropane, 6-Hydroxyhyoscyamine, Pseudotropine, 3á-Tigloyloxytropane, Hydroxy-6-tigloyloxytropane, Phenylacetoxytropane, 3-Tigloyloxy-6-(2-methylbutyryloxy) tropane, Hyoscyamine, 3-Tigloyloxy-6-isovaleroyloxy-7-hydroxytropane, Scopolamine, Tropinone, Scopine, 6-Hydroxyacetoxytropane, 3,6-Diacetoxytropane, 3-Tigloxyloxy-6-acetoxytropane, 3-Tigloyloxy-2-methylbutyryloxytropane, 3á, 6â-Ditiglotoxytropane, 3-Acetoxy-6-isobutyryloxytropan, 3-(2-Phenylpropionyloxy) tropane, Littorine, 6-Hydroxyapoatropine, 3â, 6â-Ditigloyloxy-7-hydroxytropane, 3-Tropoyloxy-6-acetoxytropane, 3,6-Dihydroxytropane, 3â-Tigloyloxytropane, 3-Tigloyloxy-6-propionyloxy-7- hydroxytropane, 3á-Apotropoyloxytropane, Aposcopolamine, 3â, 6â-Ditigloyloxytropane, 3-(3′-Acetoxytropoyloxy) tropane, 3á-Tigloyloxy-6-hydroxytropane, Tropine, 3-Acetoxytropane, 3-Hydroxy-6-acetoxytropane, 3-Hydroxy-6-methylbutyryloxytropane, 3-Tigloloxy-6-isobutyryloxytropane, Aponorscopolamine, 7-Hydroxyhyoscyamine, Meteloidine, 3â, 6â-Ditigloyloxytropane.	*Extract* asthma treatment The ethanol extracts (leaf and seed) acaricidal, repellent oviposition deterrent [125] methanol extracts antibacterial [126]. Extract anticancer activity produce vomiting, hypertension, loss of consciousness that interacts with anti-cholinergic drugs [127] ethanolic extracts anti-inflammatory activity [128]. Ethanolic extracts of leaves larvicidal and mosquito repellent activities [129]. Extract effective in countering the toxicity of the cypermethrin pesticide toxicity [130]. Antifungal activity [131]. Aqueous and organic solvent extracts broad-spectrum vibriocidal agents [132] ethanol extract of the leaves increased Serum creatinine levels, affect on biochemical and haematological parameters [133] extract treatment of organophosphate poisoning [134]	Same as effect of extract and active principles	Alkaloids (organic esters): anticholinergic agents [135] alkaloids atropine and scopolamine diarrhoea hypoactivity, liver weight loss [136]
***Cannabis sativa Linn.***	Terpenes and sesquiterpenes: Tetrahydrocannabinol (THC), Cannabidiol (CBD),α-Pinene, Myrcene, Linalool, Limonene, Trans-β-ocimene, α-Terpinolene, Trans-caryophyllene, α-Humulene, Caryophyllene-oxide [137]. Spiro compounds, viz., cannabispiran, dehydrocannabispiran, and beta- cannabispiranol, dehydrostilbenes-3-[2-(3-hydroxy-4-methoxyphenyl) ethyll-5-methoxyphenol and canniprene, acylated 0-glucoside of apigenol, 0-glycosides of vitexin, isovitexin, orientin (leaves); a-bergamotene, b-caryophyllene, trans-b-and a- farnesene isomers, a-humulene, g-elemene, a-gurjunene, b-bisabolene, b- caryophyllene-epoxide and a-bisabolol, cannabinoids, tetrahydro-cannabinol, cannabinol, n-alkanes ranging from C, to C, 2-methyl alkanes, 3-methyl alkanes, dirnethyl alkanes, a-pinene, myrcene, limonene, terpinolene, longifolene, humulene epoxides I & 11, m-mentha-1,8 (9)-dien-5-01 (essential oil from leaves and flowers); friedelin epifridelinol, N-p-hydroxy-b-phenylethy1)-p-hydroxy-trans- cinnamamide, stigmast-4-en-3-one, campest-4-en-3-one, stigmast-4,22-diene-3- one, stigmast-5-en-3b-ol-7-one, campest-5-en-3b-01-7-one, stigmast-5,22-dien- 3b-01-7-one-cannabisativine, b-sitosterol, carvone, dihydrocarvone and some unidentified bases (root); cannabigerol, cannabichromene, 1-dehydro-tetra- hydrocannabinol, cannabidiolic acid (3-methyl-6-isopropenyl-4$-pentyl-2$,6$- dihydroxy-1,2,3,6-tetrahydrobipheny1-3$-carboxylic acid), its acetate, cannaoidiol, cannabinol, tetrahydrocannabinol, trans-cinnamic acid, n-nonacosane, eugenol, guaiacol, cannabidivarin, tetrahydrocannabivarin, cannabivarichromene, L(+)- isoleucine betaine, zeatin and zeatin nucleoside, vitexin, isovitexin, orientin, acyl derivative of apigenol, N-acetyl-glucosamine, N-acetylgalactosamine, canabitriol, 22-0-glucopyranosylvitexin, 22-0-glucopyranosylorientin, orientin, cannabispirol, acetylcannabispiral, cannabicoumaronone, 9-dehydrocannabinol, cannabitetrol, isocannabispiran, cannabifuran, dehydrocannabifuran, cannabitriol, 3,5,4$-trihydroxybibenzyl, anhydrocannabisativine, cannabichromene, trans-delta-8-tetra-hydrocannabinol, 4-hydroxymethyl benzoate and 4-hydroxy-n-propyl benzoate, delta-9-tetrahydrocannabinol, dihydrostilbenes-cannabistilbenes (plant and seed) [138]	Major toxic effects of cannabis on brain and lungs.	Leaves convulsions, otalgia, abdominal disorders, malarial fever, dysentery, diarrhoea, skin diseases, hysteria, insomnia, gonorrhoea, colic, tetanus hydrophobia excessive use causes dyspepsia, cough, impotence, melancholy, dropsy, restlessness insanity. The bark tonic, inflammations, haemorrhoids and hydrocele. The inflorescence of female plant intoxicating, stomachic, soporific, abortifacient convulsions. Seeds carminative, astringent, aphrodisiac, antiemetic anti-inflammatory. The resin is smoked to allay hiccough and bronchitis insomnia, sick headaches, neuralgia, migrain, mania, whooping cough, asthma, dysuria relieving pain in dysmenorrhoea and menorrhagia	Cannabigerolic Acid: Antibiotic cannabigerol: antibiotic, antifungal, anti-inflammatory, analgesic, GABA uptake inhibitor, reduces keratinocytes proliferation in psoriasis, Effective against MRSA, cannabichromene: antibiotic, antifungal, anti-inflammatory, analgesic (weak), cannabidolic Acid: antibiotic cannabidiol: anxiolytic, antipsychotic, Analgesic, Anti-inflammatory, Antioxidant, Antispasmodic, Anti-emetic, Antifungal, Anticonvulsant, Antidepressant, Antagonizes effects of THC, Decreases sebum/sebocytes proliferation, effective against methicillin-resistant staphylococcus aureus, pro-apoptotic against breast cancer cell lines Cannabinol: Sedative, Antibiotic, Anti-convulsant, Anti-inflammatory, Decreases breast cancer resistant protein, effective against MRSA, Delta-9 tetrahydrocannabinol: Euphoriant, Analgesic, Anti-inflammatory, Antioxidant, Antiemetic, Antipruritic, Bronchodilator Delta 9 tetrahydrocannabivarin: Analgesic, Euphoriant, Anticonvulsant in vitro [139,140]
***Acorus calamus Linn.***	β-Asarone (isoasarone), α-Asarone, elemicine, cis-isoelemicine, cis and trans isoeugenol and their methyl ethers, camphene, P-cymene, β-gurjunene, α-selinene, β-cadinene, camphor, terpinen-4-ol, α-terpineol, α-calacorene, acorone, acorenone, acoragermacrone, 2-deca-4,7-dienol,shyobunones, isohyobunones, calamusenone, linalool, pre-isocalamendiol [139] Acoradin, galagin, 2,4,5-trimethoxy benzaldehyde, 2,5-dimethoxybenzoquinone, calamendiol, Spathulenol, sitosterol	Ethanolic and aqueous extract and methanolic extract antibacterial [139,141] extract asthma [142]. The ethyl acetate fraction hypoglycemic, hypolipidemia [143]. Extract inflammatory activity ethanolic extract of the rhizome: Antiulcer cytoprotective activity [144] ethanolic extract of rhizome immunosuppressive, moderately hypotensive respiratory depressant properties extract of the rhizome Antibacterial activity [145]	Leaf and rhizome antibacterial activity coconut oil extract of the rhizomes 45% inhibition of carrageenan-induced rat paw edema and 61% inhibition using the granuloma pouch method [146]	β-asarone compound fraction (crude methanolic extract of rhizomes): antifungal activity [147] Phenolic compounds: antioxidant activity [148] β-asarone and essential oil of the rhizomes: diarrhea and dysentery [149] The essential oil and alcoholic extract of the rhizomes: Analgesic activity [150] The essential oil: anti-inflammatory activity; The isolated constituted of the rhizomes, asarone and β-asarone: anticonvulsant activity [151,152] *Acorus calamus* oil induce malignant tumours, due to β-asarone in *vitro* and *in vivo* [152]

Chemical components presence in NKB may have potential interaction to produce the adverse reaction, side effects and harmful chemistry. The reported active principles may produce the probable mechanism of action of NKB and suggest the undesired herb from its chemical composition.

### The inflammatory cytokines may bind with NKB’s active principles that showed antagonistic activity

Distinctive nociceptive response is defined in early and late phases in the formalin test. The anti-nociceptive action in the formalin test of NKB paralleled with inhibitory effect of acetic acid-induced writhing. This model produces a distinct non-biphasic nociception. Drugs effect on the central nervous system can inhibit both phases equally while peripherally acting drugs inhibit the late phase [34,35]. The reason of early phase (neurogenic) may be for the stimulation of nociceptors in the paw which states centrally mediated pain while the late phase (inflammation) is due to release of serotonin, histamine, bradykinin and prostaglandins [36]. The NKB (100 mg/kg) significantly reduced compared to other samples in late phase. Formalin induced early phase and late phase inflammations is due to release of histamine and bradykinin and prostaglandin. NKB demonstrated better activity in late phase than early phase. These phases can be used to assess the potency of analgesic and the mechanisms of pain and analgesia. However, analgesic activities is different in the early (neurogenic) and late (inflammatory) phase. Acetic acid induced writhing test was used for detecting both central and peripheral analgesia. In acetic acid induced mice, acetic acid releases prostaglandins and sympathomimetic system mediators (PGE_2_ and PGF_2α_) with peritoneal fluid [37].

Abdominal constrictions shown in Figure 5D, E, in acetic acid might be related to sensitization of nociceptive receptors to prostaglandins. It is, therefore possible that the NKB exerted their analgesic effect by inhibiting the synthesis of prostaglandins. NKB exhibited significant anti- nociceptive (pain) activity as compared to control group (Figure 1C, D). NKB may show its effect through central opioid receptors or promoted release of endogenous opiopeptides.

### NKB exerted peripheral effects due to presence of flavonoids etc.

In the present study, the NKB significantly exhibited anti-inflammatory effects. As inflammation is a peripheral process, therefore, it is suggested that the NKB also exerted peripheral effects. In fact, the licking activity in the formalin test was strongly diminished in the second phase, whereas this reduction was more discreet in the first phase. Central and peripheral mechanisms may be the reason of this effect for the presence of phytochemical such as anthocyanins, proanthocyanidins, and flavonoids [30], which have potential antinociceptive and anti-inflammatory effects. Also, bioactive flavonoids such as rutin, quercetin, luteolin, hesperidin, as well as biflavonoids produce significant antinociceptive and/or anti-inflammatory activities [24,38]. Advance studies may find out the mechanisms of action of NKB and their active compounds.

NKB treated male mice showed lower effect in inflammation in the xylene induced ear edema test at dose 400 mg/kg. The NKB administered orally showed a minor inhibiting effect against ear oedema induced by xylene at the dose of 100 mg/kg. The inhibition figures were 36.36%, 51.92%, and 78.68% at the dose of 100, 200 and 400 mg/kg respectively while dexamethasone (50 mg/kg) exhibited an inhibition percentage of ear oedema of 30% as shown in Figure 1B.

### NKB formulation contains significant amount of CNS stimulant and depressant phytochemicals

The pentobarbital induced sleeping time in mice is directly proportionate to the CNS depressant activity. NKB treated female mice showed mild effect in the onset time and higher effect in the duration of sleeping time of the pentobarbital induced sleeping time test at the dose of 100 mg/kg according to control group. The onset of sleeping time is directly related to the CNS stimulant activity. It can be inferred that NKB has CNS stimulant effect that is supported to the use of NKB as CNS drug on the cerebral cortex. NKB formulation contains a lot of CNS active compounds to produce this effect (Table 3). NKB is not familiar to prescribe it as CNS stimulant medicine and hence no specific dose is found from the traditional knowledge.

The result of the present study indicates that the NKB produced a significant alteration in potentiation of pentobarbitone-induced sleeping time in a dose dependent mode. This may be due to enhancement of barbital hypnosis as a good index of CNS depressant activity [39]. NKB might have central nervous system depressant action thru its GABAergic and glycinergic transmission since pentobarbital is a selective GABAA receptor antagonist [40]. The CNS depressant activity of NKB observed in our study could be due to the presence of bufadienolide and other water soluble constituents in this herbal medicine. One of the researches on phytochemical evaluation of a medicinal plant showed the presence of bryophyllum A, B and C, a potent cytotoxic bufadienolide orthoacetate [41]. Bufadienolide was not isolated and its side effects in this study.

### NKB affected the psychological behaviors

The psychopharmacological study was carried out to find the locomotor effects of the drugs in the behavioral pattern characterized by spontaneous ambulatory activity, Centre ambulatory activity, Standing activity and emotional defecation of the animals (Figure 2). NKB treated female mice showed higher effect in total ambulatory activity at doses of 100 mg/kg *(p <0.05)* at 120 min and in 400 mg/kg. NKB treated group showed lower effect in ambulatory activity at the dose of 200 mg/kg at 120 min. NKB treated female mice exhibited higher effect in central *Ambulation* at dose 100 mg/kg at 240 min compared to the control group. NKB treated group exerted lower effect in central ambulation at doses 200 mg/kg and 400 mg/kg than 100 mg/kg (Figure 2). NKB treated female mice exerted higher effect in standing up behavior at doses of 100 mg/kg and 400 mg/kg at 120 min *(p < 0.05)* in comparison to that of the control group. NKB treated group showed lower effect in standing up behavior at dose of 200 mg/kg 60 min in comparison to that of the control group. NKB increased emotional defecation at the dose of 100 mg/kg in compared to control group and decreased at the dose of 200 mg/kg and 400 mg/kg at 60 min and 120 min and at 30 min and 60 min respectively.

### Anxiolytic activity also was observed from the psychological test method due to presence of alkaloids, flavonoids and saponins

The hole board apparatus was used in the measurement of head dipping behavioral responses of mice to an unfamiliar environment. NKB treated female mice showed an overall decrease in ambulatory activity in the hole board test at the dose of 200 and 400 mg/kg at 120 min compared to the control group (Figure 3A). NKB treated group showed an overall decrease in head dipping activity in the hole board test at the dose of 200 mg/kg (Figure 3B). NKB treated group showed higher effect in emotional defecation at the doses 200 mg/kg, and 400 mg/kg at 30 min (Figure 3C). The drug showed lower effect in the emotional defecation at the 1^st^ hour at the doses of 100 mg/kg. Decrease in the number of head dips was measured and groups that received NKB (100, 200 and 400 mg/kg) and diazepam 2 mg/kg had shown significant decrease in head dip counts. The anxiolytic effect of alkaloids [42], flavonoids [43] and saponins [44] has been previously reported and, therefore, it may be suggested for anxiolytic effects for these constitutes. Most of the anxiolytic agents exert their action by opening of activated GABAchloride channel. It is also reported that many flavonoids were found to be ligands for the GABA-A receptors in the central nervous system.

### NKB showed anxiolytic activity by modulating the GABAergic activity

NKB only for a medium dose induced a hypnotic consequence. These effects associated with NKB were similar to diazepam activity. In truth NKB showed antianxiety, sedative and lean muscle relaxation effects as being a benzodiazepine. These effects might be for the inhibitory systems like GABAergic. Flavonoids isolated from plants show to hold the affinity for GABAA/BDZ receptors as partial agonistic [38,45,46]. NKB demonstrated anxiolytic action dose dependently mainly because it increased the percentage of time spent on view arms. The reason associated with hypnotic activity might be due to binding with the phytochemicals of used plant in NKB to benzodiazepines receptors (BZ1, BZ2 and BZ3) to exhibit anxiolytic, hypnotic, lean muscle relaxation and anticonvulsant [47].

### Anti-depressant activity of NKB was found in the locomotion and plus maze test

Locomotor activity test is normally used for evaluation of central nervous system depressant and stimulant agents. It has been reported that various antidepressants like tricyclic antidepressants and monoamine oxidase inhibitors decrease locomotor activity of the mice [48,49]. In our research NKB and standard antidepressants also reduced the general locomotor behavior of the mice, at dose range that showed profound antidepressant activity in other behavioral tests. Thus, supporting that antidepressant effect of either NKB or standard antidepressants was not due to stimulation of the central nervous system. Diazepam is known for its action to inhibit aminobutyric acid type-A gamma aminobutyric acid (GABA)-activated channel [50]. It causes convulsions by inhibiting chloride ion channels associated with GABA-A receptors.

### NKB showed CNS depressant activity in hole cross study

In hole cross experiment, NKB treated mice showed lower effect at 100 and 200 mg/kg and effect was higher according to increased dose level to the 400 mg/kg & 400 mg/kg (Figure 3D). Gamma-amino-butyric acid (GABA) is the major inhibitory neurotransmitter in the central nervous system. CNS depressant drugs show their action through GABAA receptor [51]. The sedative effect of the NKB at the doses (100, 200 and 400 mg/kg, Figure 3) may be due to hyperpolarization of the CNS via interaction with GABAA or benzodiazepine receptor. The decrease in locomotion activity by diazepam treated mice compare with the control may be due to the dose (400 mg/kg) used in the test that can produce sedation in mice [52].

### NKB’s higher and lower doses increased the anti-convulsion effects in plus maze and climbing study

Almost all doses of NKB decreased locomotion activity and behavioral parameters in the elevated plus maze test in a dose-dependent manner. It is recorded that NKB have been used in reducing fear, curing trance and some other disorders in the central nervous system for instance, convulsion caused through fever and despression symptoms [53]. Herbs may lessen the motor activity and prolonged your sleeping time elicited by hexobarbital plus ameliorated ethanol-induced disability of learning along with memory [53,54]. In Climbing out experiment the decreased number of animals climbed out of the cage and increased number to come out of the cage with the time was directly proportionate to the doses (Figure 4A). NKB treated mice exerted lower effect at the dose of 400 mg/kg to come out of the cage. On low doses (100 mg/kg), NKB increased any time spent in the open-sided arms and this indicated an antianxiety effect of this herbal medicine (Figure 4B). However, this effect suggests the presence of sedative effects.

### Anxiolytic activity of NKB was found in plus maze and psychopharmacological effect in hole board study

Anxiolytic activity was evaluated using elevated plus maze paradigm and psychopharmacological activity by using hole board test. The Elevated Plus-Maze (EPM) test is used for screening anxiolytics [52]. The fear due to height induces anxiety in mice when placed on the apparatus. Mice will normally prefer to spend much of their allotted time in the closed arms. This preference appears to reflect an aversion towards open arms that is generated by the fears of the open spaces. In the present study, the total time in open arm was found to increase by the NKB (100 mg/kg). This indicates the anxiolytic activity of NKB. NKB 100 mg/kg shows significant increase in total arm entries which indicates the depression of locomotion by low dose of NKB. Decrease in movement in close arm was found at the dose 200 and 400 mg/kg. Its validity in our study was supported by the observation that diazepam, a classic anxiolytic, significantly increased the time spent in the open arms. The behavior observed using the EPM (elevated plus maze) in the present study confirmed the anxiolytic activity of diazepam. Using this test the NKB increased the percentage of time spent in the open arms. The NKB, similarly to diazepam, increased the time spent in the open arm. These results are suggestive that NKB has an anxiolytic-like effect in the plus-maze test.

### Potential significant signs are produced by NKB in swimming test

FST (forced swimming test) is usually used for evaluation of antidepressant agents. When a normal animal is forced to swim in an unsuitable area from which there is no escape, it is interested to immobility posture, reflecting a state of ‘behavioural despair’ [48]. Clinically used antidepressants have been shown to decrease the immobility time in rodents and non-rodents. NKB (100 mg/kg) treated group showed an increase in the immobile phase of the forced induced swimming test after 2 hour (Figure 4D). This effect was similar to tricyclic, monoamine oxidase inhibitors and other atypical antidepressants [48,49]. The observed U-shape (Figure 4D at 24 h) (biphasic effect) dose–response relationship in FST may be due to mixture of phytochemical compounds of used herbs in the NKB. The reduction of the duration of immobility in the swim test may be due to activating catecholaminergic mechanism in the brain [55,56]. The attenuation of immobility in FST by NKB indicates that this effect may have occurred due to increase level of catecholamine, possibly noradrenaline in the brain.

### NKB produced higher sedative effects than diazepam

NKB showed increased sedative effect similar to that observed with 3 mg/kg diazepam in the Pentobarbital induced sleeping test (Figure 5A), after oral administration of 100, 200, and 400 mg/kg dosages. Diazepam is an approved anxiolytic benzodiazepine (BDS) which produces both anxiolytic-and sedative effects. In this respect, we found a dose-dependent reduction in NKB in the number of head dips in the hole-board test similar and/or greater than diazepam.

Usually locomotor activity results from brain activation as an excitation of central neurons involving different neurochemical mechanism and an increase in cerebral metabolism. The sedative activity of NKB may be mediated by GABAergic pathway, since GABAergic transmission can produce profound sedation in mice [57]. GABA inhibits the process in the opening of chloride channels to allow hyperpolarizing the membrane, leading to CNS depression for sedative and hypnosis activity. The excitatory and inhibitory neurotransmitters are Glutamate and GABA in the mammalian brain respectively [58]. Thus, these receptors of these two neurotransmitters are targeted for psychotropic drugs.

### NKB supports its use as folk medicine due to significant duration of efficacy

In the test of pentobarbital-induced sleep in mice, NKB not only prolonged the sleeping time but also decreased the latency of falling asleep. The NKB produced hypnosis at high dose of 200 and 400 mg/kg. This results showed that NKB contains Phytochemical of coumarin, chalcones, flavanones, flavones, flavonols, quercetin, and kaempferol derivatives to work as the pentobarbital on the CNS involves the activation of the inhibition GABAergic system [59-63] (Table 3). In conclusion, administration of NKB induces similar sedative effects, supporting its use in folk medicine. It seems that that the LD50 value for this herbal drug was beyond 1000 mg/kg for oral administration. So there may be a remote risk of acute toxicity and good tolerance of the plants used in this traditional medicine of NKB which has obvious sedative and hypnotic activity and provides a pharmacological basis for its therapeutic efficacy on insomnia.

### Survival time is lower due to use of NKB

The hypoxia induced convulsion onset time is inversely proportionate to the brain oxygen demand. NKB treated male mice showed lower survival time in hypoxia test at the doses of 100 mg/Kg and no effect found in 200 mg/kg and 400 mg/kg too (Figure 5B).

### NKB affects the motor coordination in neurons

Muscle intoxicant and motor disproportion were found in the obtained results of the rotarod test. The rotarod is sensitive to drugs that affect motor coordination. The experiment was carried out to find the pattern of behavior, characterized by percentage of fall and number of falling. NKB treated female mice showed increasing effect in the total fall of the rotarod test at the dose of 400 mg/kg. This increased effect was observed only in the initial 1^st^ hour at the dose of 400 mg/kg.

### Anti-diarrheal and higher intestinal motility is found in NKB

The common side effects such as mild diarrhoea, loss of weight, depression and varied weight after 7 days were not recorded. The mice treated with doses up to 400 mg/kg of NKB exhibited significant anti-inflammatory and analgesic activities. Therefore, we tried to investigate whether this dose would provoke any genotoxic effect.

The milk meal model is used for finding the GIT motility in animal Changes in tannin content, polymerisation and models [64,65]. The significant GIT motility was protein precipitation capacity with increasing the dose of NKB (Figure 5D). The NKB is good constipating as it increased the intestinal motility.

Gastric emptying test was carried out in order to assess the effect of the NKB on the emptying of a solid meal from the gastric cavity. NKB treated male mice exerted increased effect *30%* (Lapse time) in gastric emptying at the dose of 100 mg/kg (Figure 5E). The NKB also inhibited normal gastric emptying; this effect may be linked to the reduction in gastrointestinal propulsion observed in the mice. Decrease in intestinal transit time by morphine and atropine are linked to delay in gastric emptying [66-69]. This suggests that the plant may have morphine-like action in exerting its antidiarrheal activity.

### Comparative overall effects of the NKB

A reciprocal result was found in NKB treated female mice using pentobarbital induced sleeping in CNS activity test. Higher CNS activity was found at the dose of 200 mg/kg but other doses showed little effect. The same dose (200 mg/kg/day) is used by the herbal medicine practitioner for the treatment of sleeping disorder. NKB treated male mice, showed lower metabolic effect in the survival time of the hypoxia test at the dose 100 mg/kg. NKB showed increased of writhing response at a dose of 200 and 400 mg/kg *(p <0.01)* in the Acetic Acid induced abdominal writhing test. Reverse effect was observed at the lower doses (100 mg/kg) and suggest an adjustment of doses to get proper analgesic effect. Mild analgesic and higher anti-inflammatory effect of NKB *(P < 0.05)* observed by formalin induced paw licking test and higher anti-inflammatory effect of NKB were found in xylene induced ear edema test. Neuropharmacological effects were determined using open field test of ambulation, standing up behavior, emotional defecation and hole board test, locomotor test and hole cross test those showed positive effect dose dependently. Observed psychopharmacological effects using climbing out test, elevated plus maze, showed better effect at the dose of 100 mg.kg of NKB. Observed results from toxicology study by screening the rate of gastrointestinal motility and gastric emptying rate showed opposite effect based on the use of dose. In gastric emptying, higher rate at higher doses but NKB with lower dose of 100 mg/kg *(p < 0.040)* showed an increase gastro intestinal motility. The plant *Acorus calamus Linn.* may be responsible for this effects (Table 3). The results of the present work clearly demonstrate the significant anti-inflammatory and peripheral analgesic activities of the different doses of NKB. The same medicine exhibited gastric emptying and gastrointestinal motility effects. On the other hand, NKB influence some side effects. These results indicate that NKB might have toxic dose properties that can be exploited in food and pharmaceutical industries.

## Conclusions

A comprehensive screening was performed to determine the mechanism of action of NKB formulation. Most of the herbs in the NKB showed the hypnotic, metabolic, analgesic, anti-inflammatory, psychopharamcological, anxiolytic, neuropharamcological and toxicological activity. The plant showed the respective activity was tried to correlate with the earlier published studies. It has been found that *Canscora decussata (Roxb.) Schult.* has no effect as analgesic activity. Anti-inflammatory activity found from the *Datura stramonium Linn.* but its active compounds alkaloids atropine and scopolamine may cause diarrhea, vomiting and cytotoxicity (Table 3). *Acorus calamus Linn*. may be responsible for hypnotic activity but it showed diarrhea and immunesuppressive activity (Table 3). Apart from this the metabolic activity, gut motility and gastric emptying activity may be due to these three herbs. These findings might be fruitful to develop the NKB with rational scientific and evidence based design. It is proposed that the herbs used in the NKB’s composition and their active principles with similar kind of properties need to be revised for optimum activity. In case of dose determination, 100 mg/kg/day is recommended as effective concentration of NKB to optimize the desired efficacy and safety. This strategy might be helpful for optimum pharmacological effects and safety for NKB and other herbal medicines, too.
